# Co-transfer of antigen and contextual information harmonises peripheral and lymph node cDC activation

**DOI:** 10.1126/sciimmunol.adg8249

**Published:** 2023-07-21

**Authors:** C Pirillo, S Al Khalidi, A Sims, R Devlin, H Zhao, R Pinto, S Jasim, PA Shearer, AL Shergold, H Donnelly, A Bravo-Blas, C Loney, G Perona-Wright, E Hutchinson, EW Roberts

**Affiliations:** 1 CRUK Beatson Institute, Glasgow, Great Britain; 2 MRC-University of Glasgow Centre for Virus Research, Glasgow, Great Britain; 3 School of Infection and Immunity, University of Glasgow, Glasgow, Great Britain; 4 School of Cancer Sciences, University of Glasgow, Glasgow, Great Britain; 5 Jinan Center for Disease Control and Prevention, Jinan, Shandong 250021, China

## Abstract

T cell responses against infections and cancer are directed by conventional dendritic cells (cDC) in lymph nodes distant from the site of challenge. Migratory cDC which travel from the tissue to the lymph node drive initial T cell activation but also transfer antigen to lymph node resident cDC. These resident cells have essential roles defining the character of the resulting T cell response, however, it is unknown how they can appropriately process and present antigens to suitably direct responses given their spatial separation. Here using a novel strain of influenza A and a modified melanoma model we show that tissue and lymph node cDC activation is harmonized and that this is driven by co-transfer of contextual cues. In the tumour, incomplete cDC activation in the tumour microenvironment (TME) is mirrored by lymph node resident cDC, whilst during influenza infection pathogen associated molecular patterns co-transferred with antigen drive TLR signalling in the resident cDC and their subsequent robust activation. This co-transfer mechanism explains how individual antigens can be handled distinctly by resident cDC and how signals driving poor tumoral cDC activation further impact the lymph node. Our findings clarify how tissue context dictates antigenic and, consequently, T cell fate in the lymph node.

## Introduction

To direct T cell immunity the lymph node (LN) must coordinate diverse T cell outcomes to distinct antigens; this presents an acute challenge as the lymph node is spatially separated from the site of antigen encounter. As such, to understand what determines the quality and character of T cell responses it is critical that we understand how information about antigenic context is encoded and transmitted to the LN. This information transfer is carried out by conventional dendritic cells (cDC) which detect contextual cues such as pathogen associated molecular patterns when engulfing material; these cues dictate antigen processing, expression of costimulatory molecules and cytokine production (1–3). These cDC comprise two distinct lineages of cells, cDC1 and cDC2 (4), which have been suggested to be critical for driving CD8^+^ and CD4^+^ T cell responses respectively with cDC1 being functionally specialised to perform cross-presentation of exogenously acquired antigen (5–7). While this was originally proposed it has become clear that there is considerable cooperation between these cell types with both playing important roles in the priming of both CD8^+^ and CD4^+^ T cell responses (8, 9).

Upon activation in the tissue both cDC subsets then migrate to the LN, where T cells recognising their cognate antigen receive the associated signals that direct their subsequent differentiation (10). These migratory cDC (mDC) have been shown to drive and direct CD4^+^ and CD8^+^ T cell responses in homeostasis, infection and tumour settings (11–15), with both migratory cDC1 (mDC1) and migratory cDC2 (mDC2) being critical for the initiation of robust anti-viral and anti-tumour responses. Thus mDC can explain a proportion of the information transfer occurring between the site of challenge and the lymph node, but crucially lymph node resident cDC (rDC) have critical roles in dictating the quality and quantity of a T cell response (16–20). How they receive information to appropriately direct this is unknown and so while it is understood how mDC can interpret antigenic origins there remains a large gap in our understanding of how tissue context is transmitted to dictate the priming environment and T cell fate. rDC also comprise both cDC1 (rDC1) and cDC2 (rDC2). rDC1 have been the main focus of work identifying the roles of rDC with rDC2 less well understood; rDC1 are thought to act as platforms bringing together T cells activated by mDC to facilitate T cell help (19, 20) and also allow the distribution of tissue derived antigen to a broader network ensuring more efficient T cell search (21). Given these key roles determine the quality of the downstream T cell response, an appreciation of how rDC receive antigen and interpret accompanying signals will be informative in defining the factors driving effective T cell immunity.

Currently it is know that living mDC transfer antigens to rDC (22) through a poorly defined mechanism which may involve exosomes, cross-dressing, synaptic transfer or secretion of proteins (22, 23). This allows rDC to present antigen, increasing the abundance of antigen loaded cDC and so improving search efficiency for rare antigen specific T cells (22). rDC are also essential in coordinating T cell help, driving efficient anti-viral T cell immunity, and in maintaining peripheral tolerance (19, 20, 24). These tasks involve communicating the context of antigen acquisition to the T cells, to direct the size and quality of the adaptive response; but the mechanisms by which rDC acquire contextual information, given their physical distance from the site of infection, is still unknown. Some mechanism for rDC to receive antigen contextual cues must therefore exist; hypothesised methods for this have included rDC activation by: cytokine draining from tissue (25); CD4^+^ T cell help (26) or local cytokine stimulation (27). Each of these can be considered as analogous to lossy data compression, with global activation losing all antigen specific information, and both CD4^+^ T cell help and local cytokine delivery failing to provide cues directing antigen presentation and so necessitating source agnostic antigen processing; furthermore these do not appear to explain full activation of cDC which requires sensing of pathogen derived material (28). It is currently unclear to what extent antigenic tissue context and rDC phenotype can in fact be integrated.

These different methods of harmonizing peripheral antigenic cues and rDC phenotype have different implications for how T cell responses to infectious agents and cancer may be regulated or dysregulated. Specifically these proposed hypotheses suggest that all antigens acquired by activated rDC are all treated equivalently raising questions about maintenance of peripheral tolerance during infectious challenge. Furthermore, they have implications for the design of vaccines and immunotherapies determining how rDC function could be promoted to drive more robust immune responses. In order to examine how cDC activation in the periphery relates to rDC phenotype we used ‘BrightFlu’, a novel genetically tagged influenza A virus (IAV) that causes infected cells to express ZsGreen, to track viral antigen from the lung to the LN demonstrating that rDC are specifically activated upon acquisition of viral derived antigen. Next we compared BrightFlu to a ZsGreen tagged model of experimental metastasis and saw that tumour antigen loaded rDC activation was distinct to that seen during IAV infection and that this mirrored cDC activation in the tissue, demonstrating a high degree of information co-encoded with received antigen. Information transfer was independent of previously suggested mechanisms including CD4^+^ T cell help and of type I interferon (IFN). In the IAV setting activation relied on co-transfer, and rDC sensing, of pathogen associated molecular patterns. Thus, co-transfer of antigen and contextual information allows integration of the lung and LN coordinating immunity in the context of both viral infection and tumour development.

## Results

### BrightFlu derived ZsGreen is trafficked by mDC to lymph node rDC

**Figure 1:**
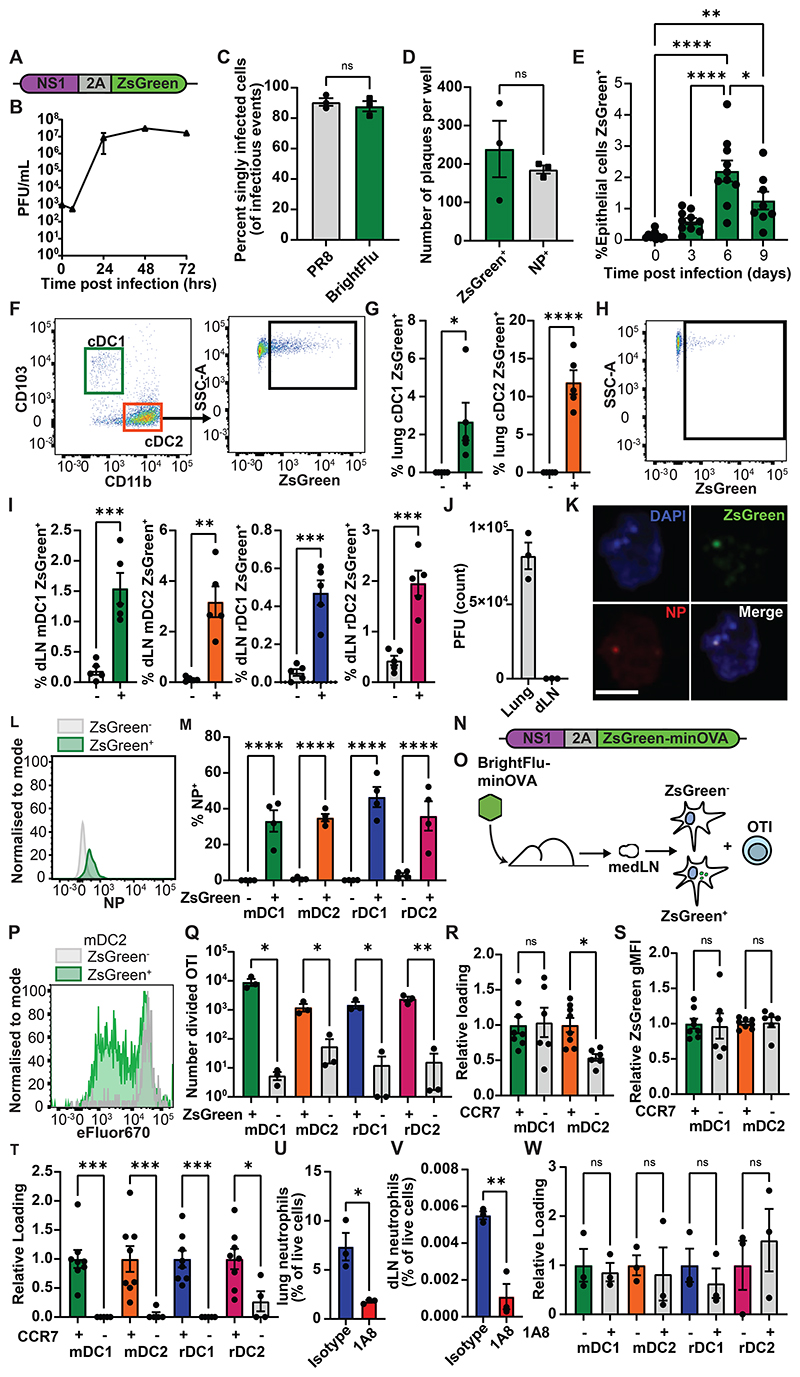
PR8 derived ZsGreen is trafficked to the dLN and transferred to rDC. (A) Schematic of modified NS1 gene segment used to generate BrightFlu (B) Multicycle growth curve of BrightFlu in MDCK cells (C) Proportion of singly infectious particles and (D) number of NP or ZsGreen expressing plaques *in vitro* (E) Time-course showing proportion of lung epithelial cells positive for ZsGreen after infection with 100 PFU of BrightFlu (n=38, 3 experiments) (F) Representative flow plots of lung cDC ZsGreen loading (G) Proportion of lung cDC positive for ZsGreen at day 6 post infection with 100 PFU of BrightFlu (n=5, 3 experiments) (H) Representative flow plot showing loading of ZsGreen in cDC of the draining lymph node (dLN) (I) Proportion of dLN cDC subsets positive for ZsGreen at day 6 post infection with 100 PFU BrightFlu (n=5, 3 experiments) (J) Plaque assay of lungs and dLN taken from mice at day 6 post infection with 100 PFU BrightFlu (n=3, 2 experiments) (K) Confocal images of sorted lymph nodes resident cDC showing ZsGreen (green), NP (red) and DAPI (blue). Scale bar = 5μm (L) Representative flow plot showing NP staining of ZsGreen^+^ (green) and ZsGreen^-^ (grey) mDC1 (M) Proportion of ZsGreen^+^ and ZsGreen^-^ LN migratory conventional (mDC) and resident conventional (rDC) subsets positive for NP (n=4 mice, 2 experiments) (N) Schematic of modified NS1 gene segment used to generate BrightFlu-minOVA (O) Schematic of experimental approach to evaluate activation of OTI cells by cDC subsets in draining LN (P) Representative flow plot showing OTI division with flow sorted ZsGreen^+^ and ZsGreen^-^ mDC2 (Q) Number of live, divided OTI T cells at 72 hours post incubation in the presence of ZsGreen^+^ and ZsGreen^-^ cDC subsets (cells sorted from 20 mice, 3 replicates per condition, 2 experiments) (R) ZsGreen loading of cDC in lungs of wildtype controls and CCR7^-/-^ mice (n=14, 2 experiments) (S) ZsGreen gMFI of ZsGreen^+^ cDC in lung of wildtype controls and CCR7^-/-^ mice (n=14, 2 experiments) (T) ZsGreen loading of cDC in medLN of wildtype controls and CCR7^-/-^ mice (n=14, 2 experiments) (U) Neutrophils as a proportion of lung and (V) dLN live cells ± 1A8 depletion (n=6, 2 experiments) (W) ZsGreen loading of LN cDC subsets in isotype treated controls and 1A8 depleted mice (n=6, 2 experiments). Statistical differences were determined by student T test (G, I, M, Q, R, S, T, U, V, W) and one-way ANOVA I. P values: ns P > 0.05; *P < 0.05; **P < 0.01; ***P < 0.001; and ****P < 0.0001. Data are shown as mean ± SEM

To generate a system where antigen could be tracked from the site of infection to the LN, a recombinant influenza virus was generated that encoded ZsGreen downstream of the viral NS1 protein, separated by a 2A linker sequence (Fig. 1A). The modified virus, BrightFlu, replicated efficiently under multicycle growth conditions (Fig. 1B), produced a comparable proportion of semi-infectious particles to unmodified PR8 IAV (Fig. 1C), and reliably expressed the ZsGreen tag (Fig. 1D), demonstrating that BrightFlu is infectious and genetically stable *in vitro. In vivo*, when mice were intranasally (i.n.) infected with 100 PFU of BrightFlu, CD45^-^ EpCAM^+^ cell infection peaked at day 6 post infection similarly to wildtype PR8 (Fig. 1E). Typical spatial patterns of infection within the lung were observed by light sheet microscopy (Fig. S1A). Critically for this study, in precision cut lung slices neutrophils could be observed that had phagocytosed viral derived ZsGreen, demonstrating the feasibility of tracking viral material (Fig. S1B). Indeed, at day 6 post-infection, cDC1 (CD103^+^) and cDC2 (CD11b^+^) in the lung had ingested viral derived ZsGreen (Fig. 1F-G, S2A) and in the draining mediastinal lymph node (medLN) migratory cDC1 (mDC1) and cDC2 (mDC2) as well as lymph node resident populations of cDC1 (rDC1) and cDC2 (rDC2) all carried ZsGreen (Fig. 1H-I, S2B). Given the reported upregulation of MHCII by rDC1 upon IAV infection (29), mDC1 and rDC1 were also identified using an MHCII insensitive gating strategy to confirm rDC1 identity (Fig. S2C). Detection of ZsGreen represented antigen transport, as viable BrightFlu viruses were found in the lung but not the medLN (Fig. 1J). To further confirm that ZsGreen^+^ cDC carried antigen rather than being infected, BMDC were infected *in vitro* or were co-cultured with ZsGreen^+^ B16F10 cells; infected cells showed cytosolic distribution of ZsGreen while those which had phagocytosed ZsGreen showed a highly punctate, vesicular distribution of ZsGreen (Fig. S3A). ZsGreen^+^ cDC in the LN were sorted and examined by Imagestream demonstrating that these all showed vesicular distribution of ZsGreen while sorted ZsGreen^+^ CD45^-^ cells from the lung predominantly showed an infected, cytosolic distribution (Fig. S3B). Finally, sorted ZsGreen^+^ cDC from the medLN were imaged by confocal microscopy and also showed the characteristic punctate staining, indicating phagocytosis rather than infection (Fig. S3C). To confirm that ZsGreen faithfully tracked viral antigens, ZsGreen^+^ rDC were sorted from the medLN and stained for viral nucleoprotein (NP) which colocalised with ZsGreen (Fig. 1K). Furthermore, by flow cytometry NP was seen to be restricted to ZsGreen^+^ cDC within the medLN (Fig. 1L-M). To further confirm that ZsGreen faithfully tracked antigen a second strain of IAV was generated with the previously published ZsGreen-minimal OVA (23) construct fused to the NS1 peptide via a 2A sequence (Fig. 1N). Mice were infected with 100 PFU of this BrightFlu-minOVA virus and at day 6 post infection ZsGreen^+^ and ZsGreen^-^ cells were sorted from each medLN cDC subset before being used to stimulate OTI T cells *in vitro* (Fig. 1O). While all sorted subsets could drive OTI proliferation after pulsing with SIINFEKL peptide (Fig. S4), similarly to as published in the tumour setting (23) only ZsGreen^+^ mDC and rDC subsets were able to stimulate OTI cell proliferation, confirming the fidelity of the ZsGreen as a tracker of antigen transfer (Fig. 1P-Q). To test whether IAV-derived ZsGreen was transported by mDC, wildtype and CCR7^-/-^ mice were infected with BrightFlu. Equivalent loading of cDC1 with ZsGreen was observed in the lung, although a lower proportion of cDC2 were loaded with ZsGreen (Fig. 1R-S). However, in the absence of CCR7 no ZsGreen reached any cDC subsets in the medLN (Fig. 1T). Furthermore, blocking chemotaxis with pertussis toxin during infection left lung loading unaffected but prevented ZsGreen from entering the rDC network (Fig. S5A-B). To confirm this was not due to neutrophil mediated transport, mice were depleted of neutrophils (Fig. 1U-V). This depletion had no impact on antigen acquisition by cDC in the lung (Fig. S5C-D), nor in the medLN (Fig. 1W). Thus, BrightFlu provides an effective model to study antigen transfer between cDC subsets *in vivo*.

### rDC activation phenotypes upon antigen acquisition mirror peripheral cDC activation

**Figure 2:**
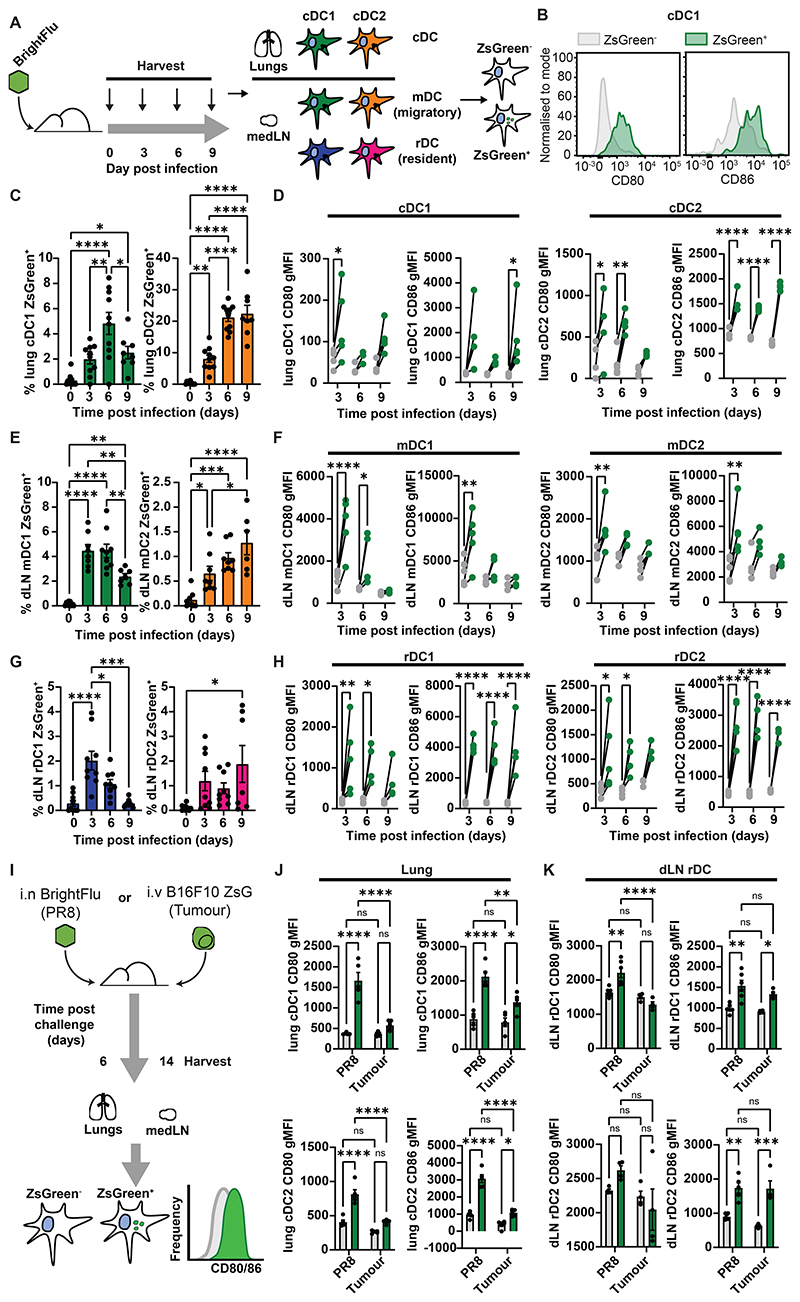
LN rDC activation mirrors cDC activation at the challenge site (A) Schematic of experimental approach to evaluate activation of conventional DC (cDC) subsets in peripheral tissue and draining LN (B) Representative flow of CD80 and CD86 on ZsGreen^+^ (green) and ZsGreen^-^ (grey) cDC (C) Quantification of antigen loading in lung cDC throughout infection with 100 PFU of BrightFlu (n=38, 3 experiments) (D) gMFI for CD80 and CD86 of antigen bearing (green) and non-bearing (grey) cDC subsets in the lung throughout infection (n=14, 3 experiments) (E) Quantification of antigen loading in draining lymph node (dLN) mDC throughout infection with 100 PFU BrightFlu (n=33, 3 experiments) (F) gMFI for CD80 and CD86 of antigen bearing (green) and non-bearing (grey) migratory cDC subsets in the dLN (n=13, 3 experiments) (G) Quantification of antigen loading in dLN resident conventional (rDC) subsets throughout infection with 100 PFU BrightFlu (n=34, 3 experiments) (H) gMFI for CD80 and CD86 of antigen bearing (green) and non-bearing (grey) resident cDC subsets in the dLN (n=14, 3 experiments) (I) Schematic of experimental approach to compare cDC and rDC activation in the context of tumour and infection (J) gMFI for CD80 and CD86 of antigen bearing (green) and non-bearing (grey) cDC subsets in the lung and (K) dLN resident cDC in BrightFlu infected and B16ZsGreen i.v. injected mice respectively (n=10, 2 experiments). Statistical differences were determined by one-way ANOVA (C, E, G, D, F), two-way ANOVA with Šidák multiple comparison test (D, F, H) or two-way ANOVA (J, K). P values: ns P > 0.05; *P < 0.05; **P < 0.01; ***P < 0.001; and ****P < 0.0001. Data are shown as mean ± SEM

To determine whether rDC activation mirrored a peripheral cDC phenotype, mice were infected with 100 PFU of BrightFlu and cDC subsets in the lung were analysed by flow cytometry (Fig. 2A-B). Both cDC1 and cDC2 acquired antigen with the percentage positive for ZsGreen peaking at day 6 (Fig. 2C), and were robustly activated upon antigen acquisition as measured by increased expression of CD80, CD86 and CD40 (Fig. 2D, S6A). mDC in the medLN showed similar antigen loading dynamics as in the tissue with mDC1 peaking earlier than mDC2 (Fig. 2E) and mDC which carried ZsGreen were activated (Fig. 2F, S6B). The rDC subsets also showed similar antigen loading dynamics (Fig. 2G) and crucially, despite being spatially separated from the challenge site, antigen loaded rDC were specifically activated in the medLN (Fig. 2H, S6B) confirming that antigen is co-encoded with contextual information. Furthermore, the activation of rDC subsets persisted beyond that of mDC subsets (Fig. 2H) suggesting that rDC may promote T cell help after initial activation. These data demonstrate that antigen acquisition is linked to rDC activation, but do not directly show that this activation contains qualitative information about the antigen source. To assess this, mice were injected intravenously (i.v.) with a modified B16F10 melanoma cell line expressing ZsGreen, which is expected to drive poorer cDC activation, or were infected i.n. with 100 PFU of BrightFlu, and their lungs analysed at day 14 post injection or day 6 post infection respectively (Fig. 2I). In the cancer setting, comparable levels of antigen loading were observed in the lung and the medLN (Fig. S7A-B), but while cDC upregulated CD80 and CD86 in response to both BrightFlu antigen uptake, only CD86 was upregulated in the cancer setting (Fig. 2J). There were similar trends in mDC, and this was not due to a difference in antigen loading (Fig. S7C-F). Critically, rDC subsets showed similar phenotypes to the lung cDC suggesting that rDC receive contextual information about antigen (Fig. 2K). In the tumour setting, transfer via infected cDC could be ruled out as antigen can only be acquired from phagocytosis of tumour material (Fig. S3A). Furthermore, cDC1 acquiring ZsGreen from tumours in the lungs also showed increased upregulation of PDL1 (Fig. S7G-H) and this was reflected in both mDC1 and rDC1 populations (Fig. S7I-J). Activation of all cDC subsets was further confirmed as ZsGreen^+^ cDC subsets from either setting all showed increased expression of IL-12 (Fig. S7K-N). This also confirmed that rDC activation was not due to cross-dressing with costimulatory molecules stripped from mDC subsets in the medLN.

### rDC antigen dependent activation is independent of CD4^+^ T cell help and type I interferon

**Figure 3:**
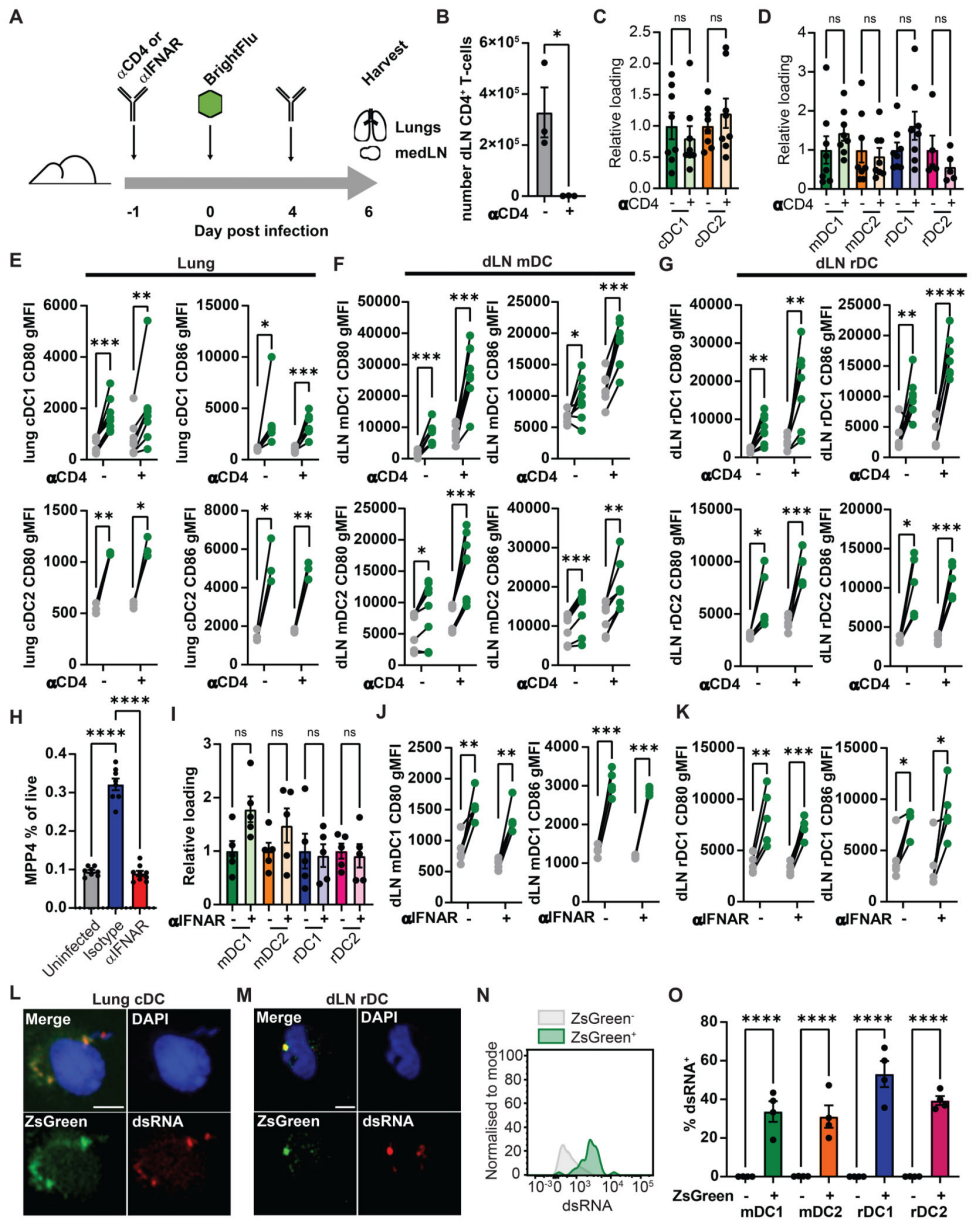
dLN resident cDC are activated independently of CD4^+^ T cell help and type I IFN signalling (A) Schematic showing blocking experiments (B) Quantification of CD4^+^ T cells in the dLN at day 6 post infection (n=6, 3 experiments) (C) ZsGreen loading of cDC in the lung and (D) dLN at day 6 post infection ± CD4^+^ T cell depletion (n=16, 3 experiments). (E-G) gMFI for CD80 and CD86 of antigen bearing (green) and non-bearing (grey) cDC subsets ± CD4^+^ T cell depletion in the lung (E), mDC subsets in the dLN (F) and rDC subsets in the dLN (G) (n=16, 3 experiments). (H) Quantification of haematopoietic multipotent progenitors as a proportion of live cells in the bone marrow of uninfected, isotype and anti-INFRα treated mice (n=25, 3 experiments) (I) ZsGreen relative loading of mDC and rDC subset in the dLN at day 6 post infection ± IFNAR blockade (n=10, 3 experiments)(J-K) gMFI for CD80 and CD86 of antigen bearing (green) and non-bearing (grey) mDC1 (J) and rDC1 (K) in the dLN ± IFNAR blockade (n=10, 3 experiments). (L-M) Confocal images of sorted lung cDC (L) and lymph nodes rDC (M) showing ZsGreen (green), dsRNA (red) and DAPI (blue). Scale bar = 5μm (N) Representative flow of dsRNA staining of ZsGreen^+^ (green) and ZsGreen^-^ (grey) cDC in the medLN (O) Proportion of ZsGreen^+^ and ZsGreen^-^ cDC positive for dsRNA staining by flow cytometry(n=4, 2 experiments). Statistical differences were determined by student T test (B, C, D, I), one-way ANOVA with Tukey post-test (H), and two-way ANOVA with Šidák multiple comparison test (E, F, G, J, K, O). P values: ns P > 0.05; *P < 0.05; **P < 0.01; ***P < 0.001; and ****P < 0.0001. Data are shown as mean ± SEM

To investigate whether previously suggested mechanisms for rDC activation could explain our observed data, we tested whether rDC activation was due to CD4^+^ T cell mediated activation or local delivery of type I IFN. To do this CD4^+^ T cells were depleted, or type I IFN receptor was blocked by antibody administration, then mice were infected with 100 PFU of BrightFlu and lungs and medLN were analysed at day 6 post infection (Fig. 3A). CD4^+^ T cells were effectively depleted by the antibody regimen (Fig. 3B), and this did not affect antigen acquisition in the lung (Fig. 3C) or in the medLN (Fig. 3D). Upregulation of CD80 or CD86 by cDC1 and cDC2 within the lung was also not affected (Fig. 3E), nor was activation of mDC in the medLN (Fig. 3F). rDC upregulation of CD80 and CD86 upon antigen uptake also did not depend on CD4^+^ T cells (Fig. 3G). Type I IFN blockade prevented IAV induced changes in haematopoiesis (Fig. 3H, S7A-C) but did not reduce uptake of antigen in the lung (Fig. S7C-D) nor its distribution in the medLN (Fig. 3I). Type I IFN signalling was dispensable for lung cDC and medLN mDC activation upon antigen uptake (Fig. 3J, S8E-F). rDC subsets too were equivalently activated in the absence of type I IFN signalling (Fig. 3K, S8G). These data suggest that neither T cell help nor local type I IFN account for rDC activation in infection. Instead, as antigen may be passed in exosomes or synaptically (23, 30) we hypothesised there may be co-transfer of contextual cues such as pathogen/damage associated molecular patterns (PAMPs/DAMPs) from the infection site. ZsGreen^+^ cDC were FACS purified from the lung and medLN of BrightFlu infected mice and stained for dsRNA known to drive cDC activation in the context of IAV (3). As expected, ZsGreen was found to colocalise with dsRNA in intracellular vesicles in lung derived cDC (3) (Fig. 3L) but strikingly this colocalization was also observed in rDC intracellular puncta (Fig. 3M) suggesting that co-transfer of antigen with accompanying pathogen associated molecular patterns occurs during infection. By flow cytometry it could be seen that dsRNA was restricted to the ZsGreen^+^ cDC suggesting that this co-transfer appears to be tightly connected (Fig. 3N). Such double acquisition of antigen and contextual cues may account for the rapid, selective activation of antigen ingesting rDC in the medLN. Together these data demonstrate that the selective activation of antigen bearing rDC cannot be explained by interferon or T cell help but co-transfer of dsRNA provides a potential alternative mechanism.

### Co-transfer of poly(I:C) with tumour derived antigen by mDC increases rDC activation

**Figure 4:**
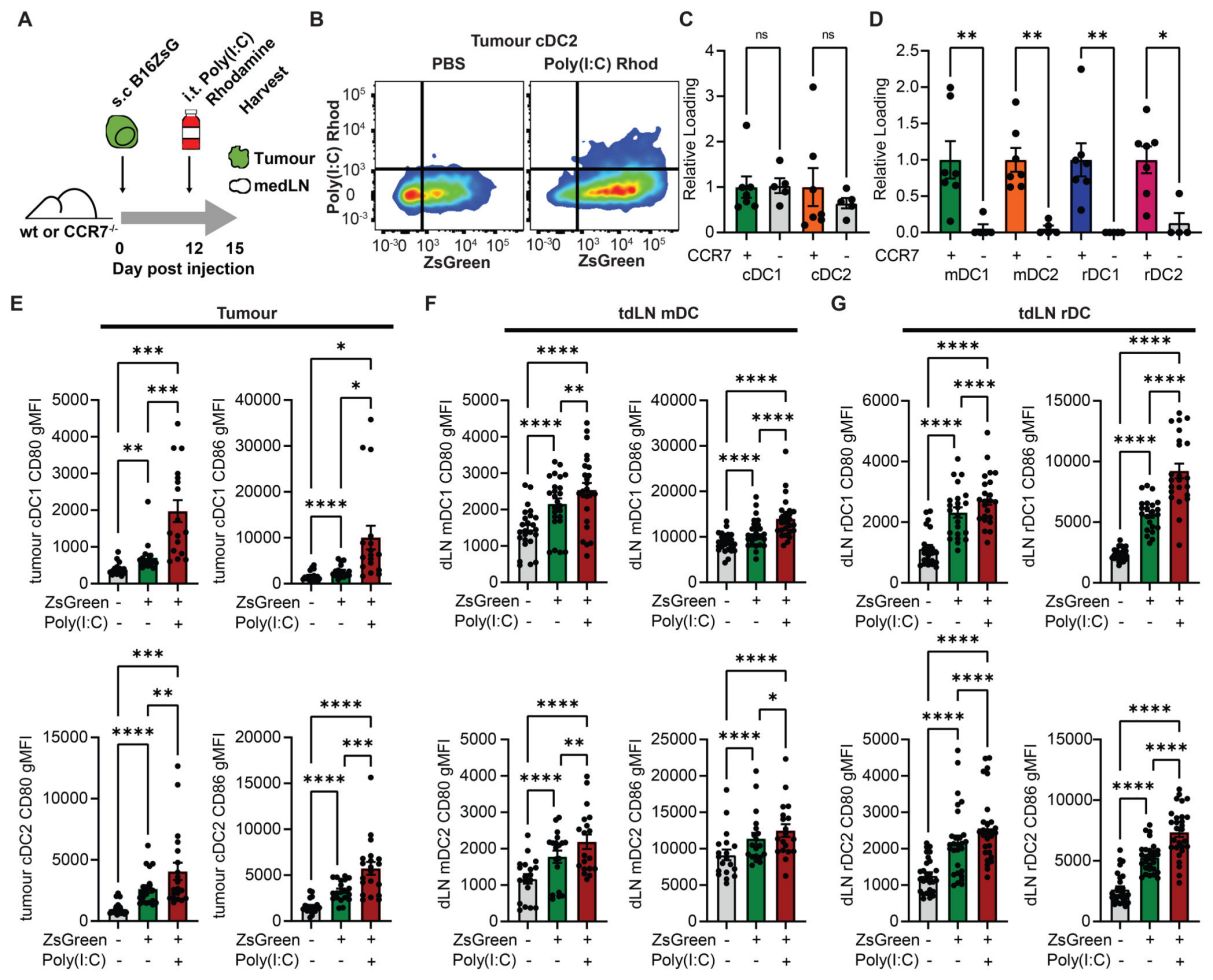
Transferred TLR ligands specifically activate recipient dLN resident cDC (A) Schematic of experimental approach taken in (B-G) (B) Representative flow plots of tumour cDC2 ± intratumoural administration of poly(I:C) rhodamine (C-D) Quantification of poly(I:C) rhodamine loading in intratumoural (C) and lymph node (D) cDC subsets in wildtype control or CCR7^-/-^ mice (n=12, 2 experiments) (E) CD80 and CD86 levels on cDC1 and cDC2 categorised by their acquisition of ZsGreen or poly(I:C) rhodamine within the tumour (n=40, 3 experiments). Too few rhodamine^+^ ZsGreen^-^ cells were present to analyse. (F-G) CD80 and CD86 levels were assessed on mDC (F) and rDC (G) categorised by their acquisition of ZsGreen or poly(I:C) rhodamine within the dLN (n=50, 3 experiments). Statistical differences were one-way ANOVA with Tukey post-test (C, D, E, F, G). P values: ns P > 0.05; *P < 0.05; **P < 0.01; ***P < 0.001; and ****P < 0.0001. Data are shown as mean ± SEM

To determine whether the transfer of PAMPs to rDC was sufficient for their activation and whether these were co-trafficked by mDC, wildtype or CCR7^-/-^ mice were injected with subcutaneous B16ZsGreen tumours. Once tumours were palpable mice were injected intratumorally with fluorescent poly(I:C). and the tumour and draining lymph node (dLN) analysed (Fig. 4A). A proportion of ZsGreen^+^ cDC in the tumour and lymph node were found to have acquired the poly(I:C), and within the tumour there was no significant difference in loading between the wildtype and CCR7^-/-^ (Fig. 4B-C). In the absence of mDC migration poly(I:C) did not reach the node, indicating that there was no passive drainage and that poly(I:C) was also trafficked to the node by mDC (Fig. 4D). Co-transfer of poly(I:C) was accompanied by activation with poly(I:C)^+^ ZsGreen^+^ cDC1 and cDC2 showing significant cell-intrinsic upregulation of CD80 and CD86 (Fig. 4C-D). Poly(I:C)^+^ ZsGreen^+^ mDC1 and mDC2 showed similar increased activation (Fig. 4E), and critically ZsGreen^+^ rDC which co-acquired poly(I:C) were also specifically activated (Fig. 4F). Thus, poly(I:C) co-transfer was sufficient to intrinsically activate rDC.

### MyD88 is required in rDC for full activation in response to transferred material

**Figure 5:**
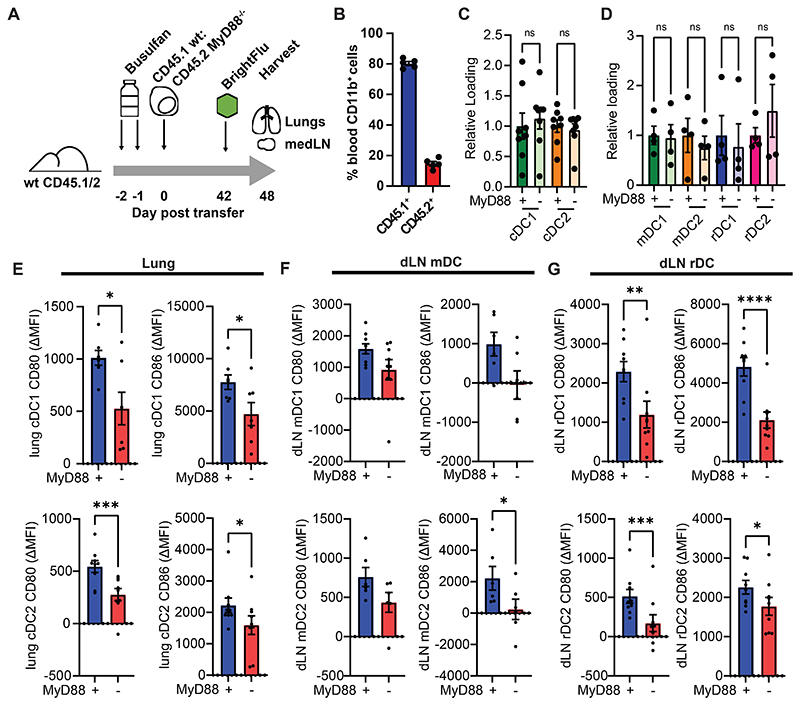
rDC require MyD88 in order to become fully activated in response to IAV antigen acquisition (A) Schematic of experimental approach taken in (B-G) (B) Percent of peripheral CD11b+ cells which were CD45.1^+^ or CD45.2^+^ at 6 weeks post bone marrow transfer (n=5, 3 experiments) (C-D) Percent of cDC in the lung (C) and dLN (D) positive for ZsGreen was assessed by flow cytometry amongst MyD88^-/-^ and wildtype cDC (n=10, 3 experiments) (E-G) Differences in CD80 and CD86 were quantified upon acquisition of viral derived ZsGreen in both wildtype and MyD88^-/-^ cDC within the lung (J), and within the mDC (K) or rDC (L) in the dLN (n=18, 3 experiments). Statistical differences were determined by student T test (E-G) and one-way ANOVA with Tukey post-test (C, D). P values: ns P > 0.05; *P < 0.05; **P < 0.01; ***P < 0.001; and ****P < 0.0001. Data are shown as mean ± SEM

To determine whether TLR ligands are also necessary for rDC activation, mixed bone marrow chimeras were generated in which approximately 20% of haematopoietic cells lacked the signalling molecule MyD88. These mice were infected with BrightFlu and the lungs and medLN analysed (Fig. 5A-B). In these mice the majority of cDC were capable of TLR signalling and so the majority of mDC would be expected to transfer any other activating signals to rDC whilst specific rDC would be unable to directly signal through MyD88 (Fig. 5B). Wildtype and MyD88^-/-^ cDC ingested antigen at comparable rates in both the lung and medLN (Fig. 5C-D). As expected, both MyD88^-/-^ cDC1 and cDC2 in the lung exhibited blunted activation upon ingestion of ZsGreen (Fig. 5E) and this trend was retained in mDC subsets (Fig. 5F). MyD88^-/-^ rDC also failed to become fully activated upon antigen acquisition (Fig. 5G) despite the majority of surrounding mDC being MyD88^+/+^ and responding normally, confirming that MyD88 signalling drives rDC activation upon antigen transfer. Together these data demonstrate that transfer of TLR ligands is necessary and sufficient to activate rDC in a cell-intrinsic manner providing a mechanism by which peripheral and lymph node cDC activation can be integrated.

## Discussion

Intercellular antigen transfer between cDC subsets has important roles in driving effective immune responses (16, 18, 23, 30). Here we demonstrate that antigen is co-encoded with contextual information and that co-transfer allows rDC to be appropriately activated in the LN despite remaining distal to the challenge site. We show in the context of IAV infection that viral antigen is co-transferred with pathogen associated molecular patterns that signal directly to recipient rDC. These findings are consistent with previous work identifying the existence of antigen transfer mechanisms such as exosomes (22), gap junctions (31) and synaptic transfer (23) all of which offer the potential for co-transfer of accompanying signals. Indeed it has been shown that multiple RNA species can be packaged into exosomes (32, 33), and when these are of viral origin they have been shown to be capable of activating recipient cDC (34). Furthermore, the presence of TLR ligands in antigen bearing phagosomes directs antigen fate (1) confirming the presence of both signals within individual compartments. Our study defines how the combination of these mechanisms leads to lossless information transfer in the immune response and demonstrates how co-encoded contextual cues and antigen allow rDC to appropriately direct T cell differentiation in these situations.

It has also been shown previously that there is selectivity in which antigens are transferred to the rDC network with ZsGreen originating from normal epidermal cells being trafficked to the LN by mDC but not being transferred (23). Given the reported importance of rDC in the process of tolerance (24) it will be important to elucidate the mechanisms by which antigen transfer occur to better understand this restriction. It will be of interest to determine whether signals driving transfer are intrinsic to the vesicle being transferred and so could represent the same contextual cues that ultimately drive rDC activation or whether they are separate. These different mechanisms could have important implications for the design of cancer therapies in determining how adjuvant therapies may be most effectively utilised to improve the priming environment in the LN.

In the context of viral infection, co-transfer allows rDC to co-ordinate T cell help, but the regulatory cDC phenotype of the TME (35) is also communicated to the rDC network of the lymph node, explaining the poor priming observed from rDC1 cells in a tumour context (23). Interestingly intratumoral poly(I:C) administration led to activation of a proportion of cDC within the TME and this corresponded to an increase in activation of both mDC and rDC in the draining LN. This may indicate that the synergistic effects seen of intratumoral poly(I:C) with checkpoint blockade and BRAF inhibition in mouse models of melanoma may be at least partially due to the increased activation of antigen bearing mDC and rDC within the lymph node (36). It would, however, be important to determine with the increased injection volume within that study whether there was direct drainage of poly(I:C), which would potentially lead to broader rDC activation than seen in this study where co-transfer was dependent on mDC migration.

The co-transfer of activating signals with antigen also provides an explanation for how rDC can become fully activated when this has been shown to depend upon direct pathogen derived material sensing (28). Interestingly, tracking ZsGreen in the setting of IAV infection demonstrated prolonged presence of antigen within cDC2 as compared to cDC1 within the lung, and in both migratory and resident subsets within the medLN. The mechanism by which this differential antigen retention occurs, or indeed whether the longer lived antigen persists within a subset of cDC2 or potentially contaminating longer lived macrophages, would be interesting to further investigate as it has previously been shown that CD4^+^ T cells rely on prolonged antigen presentation to become fully activated, whereas CD8^+^ T cells can be activated much more rapidly (37). Furthermore, antigen persistence has been proposed to play a role in maintaining effective memory responses (38) and so extending these studies to later time points could be informative about the role of different cDC subsets in antigen retention.

This mechanism of co-transfer of accompanying signals may also explain why particulate vaccines, in which adjuvant and antigen are combined for uptake by cDC, have an increased efficacy in driving T cell immunity compared to soluble vaccines where adjuvant and antigen may be taken up separately (39). This also may shed light on the failure of subcutaneous injection of synthetic TLR3 ligands to improve anti-tumour immune responses whilst intratumoral injections led to significant responses and reduced tumour growth (40). It will be interesting looking forward to determine whether this mechanism applies to other pathogen associated molecular patterns which may be less likely to be taken into endosomal compartments; for example, signals detected by plasma membrane localised TLRs such as TLR2 and 4 may be less tightly co-transferred with antigen given the potential for activation outside of endosomes. It is likely, however, that antigen acquired from bacteria and other pathogens will be accompanied by these TLR ligands, so whether these can be detected after co-transfer is another important question raised by this work. As such our findings provide a new model for the control of T cell immunity with broad implications for the design of vaccination and immunotherapy approaches.

## Materials and methods

### Study design

The aim of this study was to highlight how immune responses are coordinated appropriately at distance in dLN by cDC. ZsGreen was tracked through the cDC network originating from a newly generated PR8 influenza strain or a modified B16 melanoma to compare the activation status of cDC in the tissue and the dLN. Using flow cytometry we confirmed that viral antigen is transported to the dLN and transferred to rDC by mDC and that rDC activation mirrored that of peripheral cDC. *In vitro* T cell stimulation assays demonstrated that only ZsGreen bearing cDC activated CD8^+^ T cells illustrating the fidelity of the model. *In vivo* treatment with α−CD4 and α−IFNAR was used to test the dependence of rDC activation on these factors. Sorted ZsGreen^+^ cDC were imaged to demonstrate colocalization between ZsGreen and dsRNA. Intratumoural delivery of fluorescent poly(I:C) was used to track how PAMPs could alter the rDC activation phenotype in the tumour context in a cell intrinsic manner. Bone marrow chimeric mice were finally used to investigate the requirement for MyD88 in rDC.

### Animals and experimental design

All animal work was in accordance with the animal ethics and welfare committee at the University of Glasgow and UK Home Office regulations (ASPA, 1986, PPL P72BA642F). All mice were bred and housed at the Beatson Cancer Research Institute. C57BL/6J mice were purchased from Charles River (United Kingdom). CCR7^-/-^, OTI, CD45.1 and MyD88^-/-^ mice were purchased from the Jackson Laboratory (United States). In all experiments, male mice > 6 weeks old were used. C57BL/6, CCR7^-/-^ or MyD88^-/-^ chimeric mice were intranasally infected with 100 PFU of BrightFlu or intravenously (i.v.)/subcutaneously (s.c.) injected with 5x10^5^ B16ZsGreen cells. If not otherwise specified, for BrightFlu experiments and i.v./s.c. B16ZsGreen injected mice, endpoints were 6 days post infection and 14 days post injection respectively.

### Mixed bone marrow chimeras

Mice were injected intraperitoneally (i.p.) with 15mg/kg Busulfan for 2 days and the following day 1x10^6^ whole BM cells from donor mice were transplanted i.v. Peripheral blood reconstitution was assessed by flow cytometry 6 weeks post transplantation.

### *In vivo* treatment

For blocking antibodies, mice were injected i.p. with 250µg of α−CD4 (GK1.5, BioXcell) or 200µg of α-IFNAR (MAR1.5A3, BioXCell) one day before receiving 100 PFU of BrightFlu followed by a second dose at day 4 post infection. For depleting antibodies, mice were injected i.p. with 250µg of α-Ly-6G (1A8, Biolegend) daily for 5 days starting from 1 day prior to BrightFlu. Poly(I:C) was injected intratumorally with 10µg of Poly(I:C)-Rhodamine in 10µl PBS. For intracellular IL-12 staining, mice were injected i.v. with 250µg of Brefeldin A (Invivogen) and tissues harvested 6hr post injection.

### BrightFlu and B16ZsGreen experimental model

The sequence of NS1-2A-ZsGreen-2A-NEP was synthesised (GeneArt, Invitrogen) and cloned into the BsmBI site of the pHH21 plasmid. Recombinant virus was subsequently generated by reverse genetics.

B16F10 cells were modified to express ZsGreen as previously described (14). Cells were cultured at 37°C with 5% CO_2_ in DMEM (Gibco) containing 10% FBS, 1% Penicillin/Streptomycin (Gibco), and 1% of L-Glutamine (Gibco).

For s.c. injections, B16ZsGreen cells were lifted using 2.5% trypsin (Gibco) then washed 3 times with PBS. Harvested cells were mixed at a 1:1 ratio with Matrigel Matrix (BD Biosciences) in a final injection volume of 50µl. For i.v. injection, B16ZsGreen cell were harvested using Accutase (Stemcell technologies) and washed 3 times with PBS. Cells were then resuspended in PBS and injected in a final volume of 100µl.

### Semi-infectious particle (SIP) and NP assay

BrightFlu virus was diluted in viral growth media (VGM, DMEM containing 0.14% BSA (Gibco) and 1µg/µl TBCK-treated trypsin between the ranges of 10^-1^–10^-6^. Virus was then used to infect confluent monolayers of Madin-Darby Canine Kidney (MDCK) cells (a kind gift from Prof. P Digard, Roslin Institute, University of Edinburgh) in 6 well plates. Following a 1hr incubation, the inoculum was removed and an overlay containing 1% low melt agarose (Sigma) in VGM was added to the cells. 24hr later, the overlay was removed, cells fixed in 4% formaldehyde (Sigma) in PBS and permeabilised in 0.1% Triton-X 100 (Sigma) in PBS.

After blocking with 2% FBS in PBS, cells were incubated with rabbit α−NP antibody (a kind gift from Prof. P Digard, Roslin Institute, University of Edinburgh) then washed in PBS before staining with goat anti-rabbit Alexa Fluor 555 (1:500,ThermoFisher) and DAPI (ThermoFisher) in 2% FBS. The Celigo Imaging Cytometer (Nexcelom) was used to image the plates and quantify the number of singly infected foci and plaques for each well.

### Tissue digestion and flow cytometry

LN were collected in PBS, split open using forceps and, placed in 1ml of RPMI containing 200ug/ml DNase I (Roche), 100U Collagenase I (Worthington Biochemical) and 500U of collagenase IV (Worthington Biochemical) and incubated for 15 minutes at 37°C. Samples were triturated and incubated for a further 15 minutes at 37°C. Samples were washed with RPMI-1640 containing 10% FBS and filtered through a 70µm Nytex filter. Cells were then stained for flow cytometry.

Lungs and subcutaneous tumours were collected in PBS and transferred into GentleMax tubes containing 3ml of 10µg/ml Dispase (Gibco) and 200µg/ml DNAse I (Roche) in 3ml RPMI. Tissue was dissociated using the GentleMACS (Miltenyi) following manufacturer’s instructions. 25ml of FACS buffer (2% FBS, 0.05% NaN_3_ in PBS) was added to samples and this was then filtered through a 70µm Nytex filter. Samples were incubated in red blood cell lysis buffer (500ml dH_2_O, 0.5g KHCO_3_, 4g NH_4_Cl, 5ml 10nM EDTA, 25ml FBS) for 5 minutes at RT then washed with FACS buffer and stained for flow cytometry. Full antibody details can be found in Table S1. To label antibodies, both anti-NP(D67J, Invitrogen) and anti-dsRNA (J2, Nordic MUBio) antibodies were conjugated using the Mix-n-Stain™ CF™ 555 Antibody labelling Kit (Sigma) or the Mix-n-Stain™ CF™ 647 Antibody labelling Kit (Sigma).

Flow cytometry was performed using a LSRFortessa (BD Biosciences). Cell sorting was performed using a FACSAria II (BD Biosciences). Analysis of flow cytometry data was done using FlowJo (Treestar).

### Immunofluorescence and imaging

For sorted cDC, cells were affixed to slides using a Cytospin (Thermo Fisher Scientific) following manufacturer’s instructions, fixed for 10 minutes at RT with PLP buffer (for 10ml: 2.5ml of 4% PFA, 0.0212g of NaIO_4_, 3.75ml of 0.2M L-lysine in water, and 3.75ml of P buffer [81ml of 0.2M Na_2_HPO_4_ (Merck), 19ml of 0.2M NAPO_4_ (Merck) and 100ml of water]) and then washed 3 times with PBS. Cells were permeabilised for 10 minutes in 1% Triton-X 100 in PBS then blocked for 30 minutes in blocking solution (Bloxall). α-dsRNA (1:200, J2, Nordic MUbio) and α-NP (1:20, D67J, Invitrogen) antibodies in blocking solution (Abcam) were added to slides and incubated overnight at 4°C. Slides were washed 3 times with PBS then stained with Alexa Fluor 647 or PE conjugated secondary antibodies (1:300, Invitrogen), in protein block solution for 2.5hr at 4°C. Slides were then washed 3 times with PBS before 50µm of Vectashield anti-fade mounting medium containing DAPI (Vector Laboratories) was added and slides covered using an appropriate coverslip. Images were acquired using an LSM 710 upright confocal microscope (Zeiss).

For tissue sections, agarose inflated lungs were sectioned at 300µm using a vibrotome (5100mz Campden Instruments) and stored at 4°C in PBA (1% BSA, 0.05% NaN_3_ in PBS). Sections were permeabilized for 1hr at RT in permeabilization buffer (1% BSA, 0.3% Triton X-100, 0.05% NaN_3_ in PBS) followed by a 30 minute incubation with protein block (Abcam). Primary antibodies (1:400) in antibody diluent reagent solution (Life technologies) were added and slices incubated overnight at 4°C. Slices were washed 5 times with wash buffer (1% BSA, 0.1% Triton X-100, 0.05% sodium azide in PBS) and stained with anti-rabbit Alexa Fluor 594 (1:5000, Abcam) and/or anti-hamster Alexa Fluor Cy3 (1:2000, Abcam) for 2hr and washed as before. Ce3D tissue clearing solution (Biolegend) was then added for 30 minutes before mounting in a seal-frame incubation chamber (Thermo Scientific) covered with a coverslip. Images were acquired using Zeiss LSM 880 Airyscan confocal microscope using lambda acquisition mode.

Whole lungs were imaged by light sheet microscopy. The specimens were fixed with PLP and cleared in ethyl cinnamate (Sigma) for 4 days before excess moisture was removed and the lungs were mounted on a Zeiss mounting stub using Pattex Ultragel Superglue. The mounted specimens were then immersed in ethyl cinnamate and imaged with a Zeiss Z.1 light sheet microscope using dual-sided illumination. A 488nm laser was used and emitted light of 505-545nm was collected. The objective lens used was a Clr Plan-Neofluar 20x/1.0 Corr nd=1.45. Multiple Nyquist-optimised Z stacks were collected using Zeiss Zen software.

### Image processing and quantification

Zen black (Zeiss) was used to stitch three-dimensional (3D) lung tile scans and to unmix channels. ImageJ and Imaris (Bitplane) were used to visualize and process raw data. For light sheet imaging Z stacks were aligned and stitched into 3D montages using Imaris Stitcher (Bitplane).

### Isolation and culture of BMDC

Bone marrow was isolated from tibias and femurs and 2x10^6^ cells were resuspended in 7ml R10 (RPMI containing 10% FBS (Gibco), 1% Penicillin/Streptomycin (Gibco), 1% L-Glutamine (Gibco), 1% Hepes (Gibco), 1% NEAA (Gibco), 50mM 2-Mercaptoethanol (Thermo Fisher Scientific), 200ng/ml of FLT3L (made in house) and 10ng/ml of GM-CSF (Peprotech)) and incubated at 37°C with 5% CO_2_. After 3 days 10ml of fresh media was added then cells were resuspended in fresh media at day 6.

For *in vitro* loading, 2x10^4^ BMDCs were either infected for 1hr at 37°C with the BrightFlu virus at a multiplicity of infection of 1 or co-cultured with 2x10^4^ B16ZsGreen cells for 24hr. Cells were then washed 3 times with PBS and resuspended in fresh R10 medium. For microscopy experiments, infected or tumour fed B16ZsGreen BMDC were added to a fibronectin-coated slide, fixed for 10 minutes using 4% PFA at RT, then stained with DAPI. Images were acquired using a Zeiss LSM 880 confocal microscope.

### *In vitro* T cell proliferation assay

OTI T cells were isolated using MojoSort mouse CD8^+^ T cell isolation Kit (Biolegend) and stained with 2µM eFluor670 (Thermo Fisher Scientific). Mice were infected with 100 PFU of PR8 expressing ZsGreen-minOVA. At 6 days post infection medLN were harvested and processed as previously described. Indicated cDC subsets were sorted and 2x10^3^ cells were incubated with 2x10^4^ OTI T cells in R10. For positive controls, 2x10^3^ cDC were pulsed with 1mg/ml of SIINFEKL peptide (Invivogen) before addition to T cells. Cells were plated in a 96-well V-bottom plate for 72hr at 37°C with 5% CO_2_, and eFluor670 dilution assessed by flow cytometry.

### Statistical analysis

Data are from (N ≥ 3 or in a few cases, N=2) independent experiments. Statistical analyses were performed using GraphPad Prism software. Error bars represent mean ± SEM. Normality of data was tested using GraphPad Prism. For two groups statistical differences were determined by paired or unpaired Student’s t-test and for more than two groups, one-way ANOVA or two-way ANOVA were used as indicated. For multiple groups, Tukey post-test and Sidák multiple comparison test were used to make specific pairwise comparisons.

## Supplementary Material

Supplementary Materials

## Data Availability

All data needed to evaluate the conclusions in the paper are present in the paper or the Supplementary Materials.

## One sentence summary

Migratory dendritic cells transfer both antigen and contextual cues to lymph node resident cDC allowing their appropriate activation
