# Supplementary material for: Co-transfer of antigen and contextual information harmonises peripheral and lymph node cDC activation
Source: Sci Immunol. Author manuscript; Available in PMC 2024 May 29. (PMC7616026; doi:10.1126/sciimmunol.adg8249)
Supplement: Supplementary Materials [file EMS196319-supplement-Supplementary_Materials.pdf]

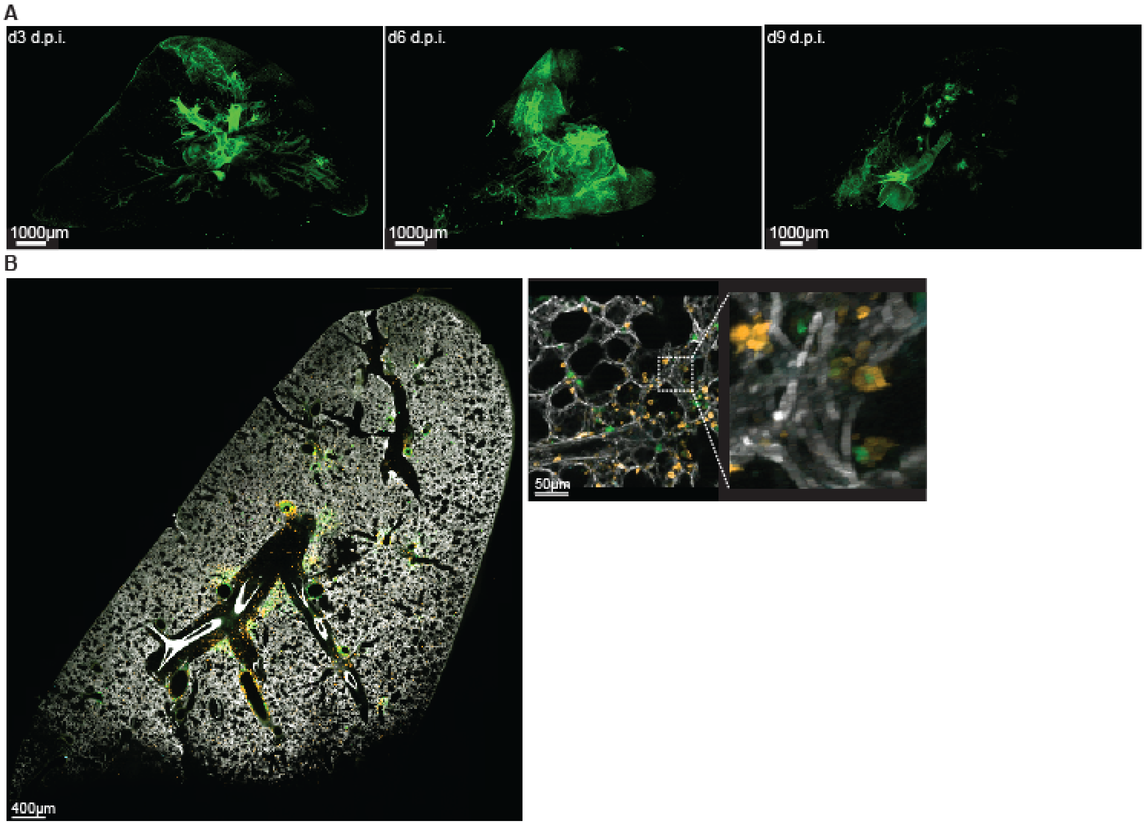

Fig. S1. BrightFlu infects regionally and ZsGreen is engulfed by neutrophils

(A) 2% agarose inflated middle lobes were harvested at indicated timepoints post infection with 100pfu BrightFlu, cleared in ethyl cinnamate and imaged by light sheet microscopy. Scale bar = 1mm (B) 2% agarose inflated left lungs were harvested at day 3 post infection with 100pfu BrightFlu, and 200µm sections cut by vibratome. Sections were stained for CD3, S100A9 and CD31 before clearing with Ce3D solution. Sections were imaged by confocal microscopy.

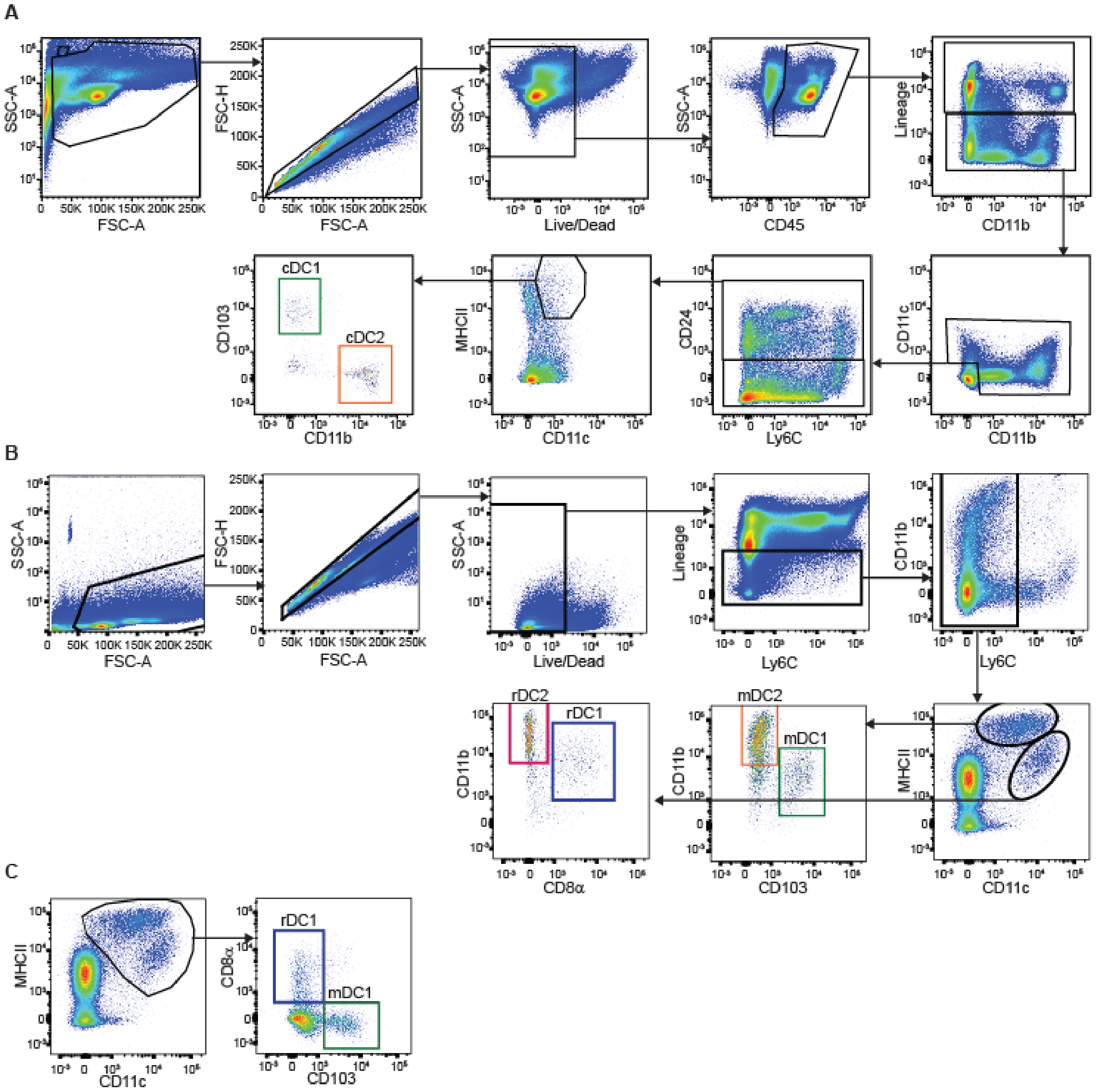

Fig. S2. Representative flow gating for cDC in lung and LN

(A-B) Representative flow of cDC gating in lung (A) and LN (B) Also alternative MHCII insensitive gating of mDC1 and rDC1 to confirm identity (C).

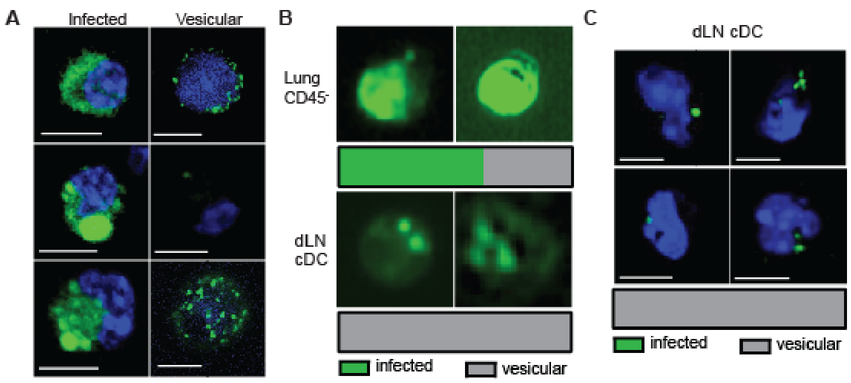

Fig. S3. ZsGreen+ cDC have acquired antigen from infected cells and are not themselves infected
(A) BMDC were infected with BrightFlu *in vitro* or incubated with B16-F10 ZsGreen tumour cells and imaged by confocal microscopy. Scale bar = 10µm (B) Sorted lung $CD45^{-}ZsGreen^{+}$ cells and LN $ZsGreen^{+}$ cDC were analysed by Imagestream. Bars display proportion of cells examined with cytosolic of vesicular distribution of ZsGreen (C) Sorted LN $ZsGreen^{+}$ cDC were analysed by confocal microscopy. Bars display proportion of cells examined with cytosolic of vesicular distribution of ZsGreen.

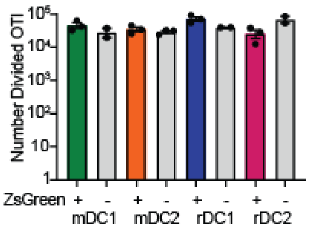

Fig. S4. SIINFEKL pulsed sorted cDC subsets are all able to stimulate OTI proliferation

Quantification of live, divided OTI T cells activated by Sorted ZsGreen$^+$ and ZsGreen$^-$ cDC subsets from LN pulsed with SIINFEKL for 72 hours. Cells were sorted from n=20 mice, 3 replicates for condition

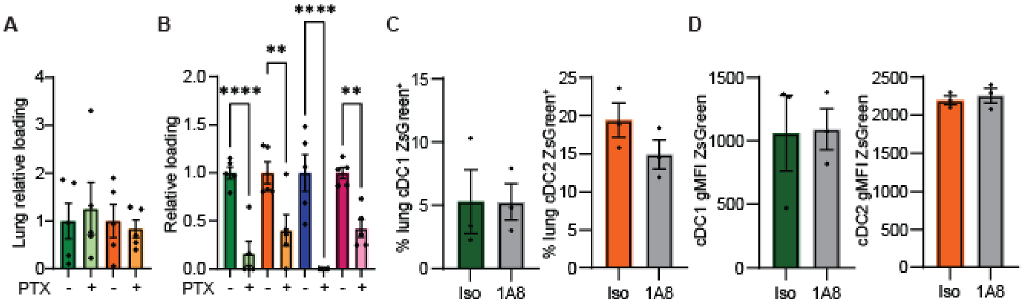

Fig. S5. PTX treatment blocks antigen transfer to rDC in a neutrophil independent manner

(A-B) ZsGreen loading of lung cDC (A) and LN cDC subsets (B) were assessed by flow cytometry at d6 post infection with 100pfu BrightFlu in the presence or absence of PTX (n=10, 2 experiments). (C-D) Lungs were harvested from mice at d6 post infection with BrightFlu +/- neutrophil depletion and the percentage of cDC subsets loaded with ZsGreen was analysed by flow cytometry (C) and the level of ZsGreen carried by loaded cDC was assessed by measuring gMFI (n=6, 2 experiments) (D). Statistical differences were determined by student T test. P values: $^*P < 0.05$ and $^{**}P < 0.01$. Data are shown as mean ± SEM

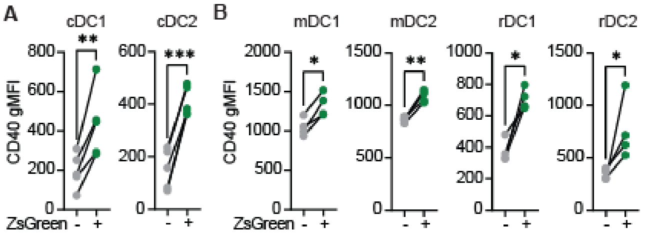

Fig. S6. CD40 is upregulated by cDC subsets upon acquisition of ZsGreen

Lungs were harvested from mice at d6 post infection with BrightFlu and the gMFI of ZsGreen in ZsGreen$^+$ (green) and ZsGreen$^-$ (grey) cDC from the lung (A) and LN (B) (n=10, 2 experiments) Statistical. Statistical differences were determined by student T test. P values: *P < 0.05, **P < 0.01 and *** P<0.001. Data are shown as mean ± SEM

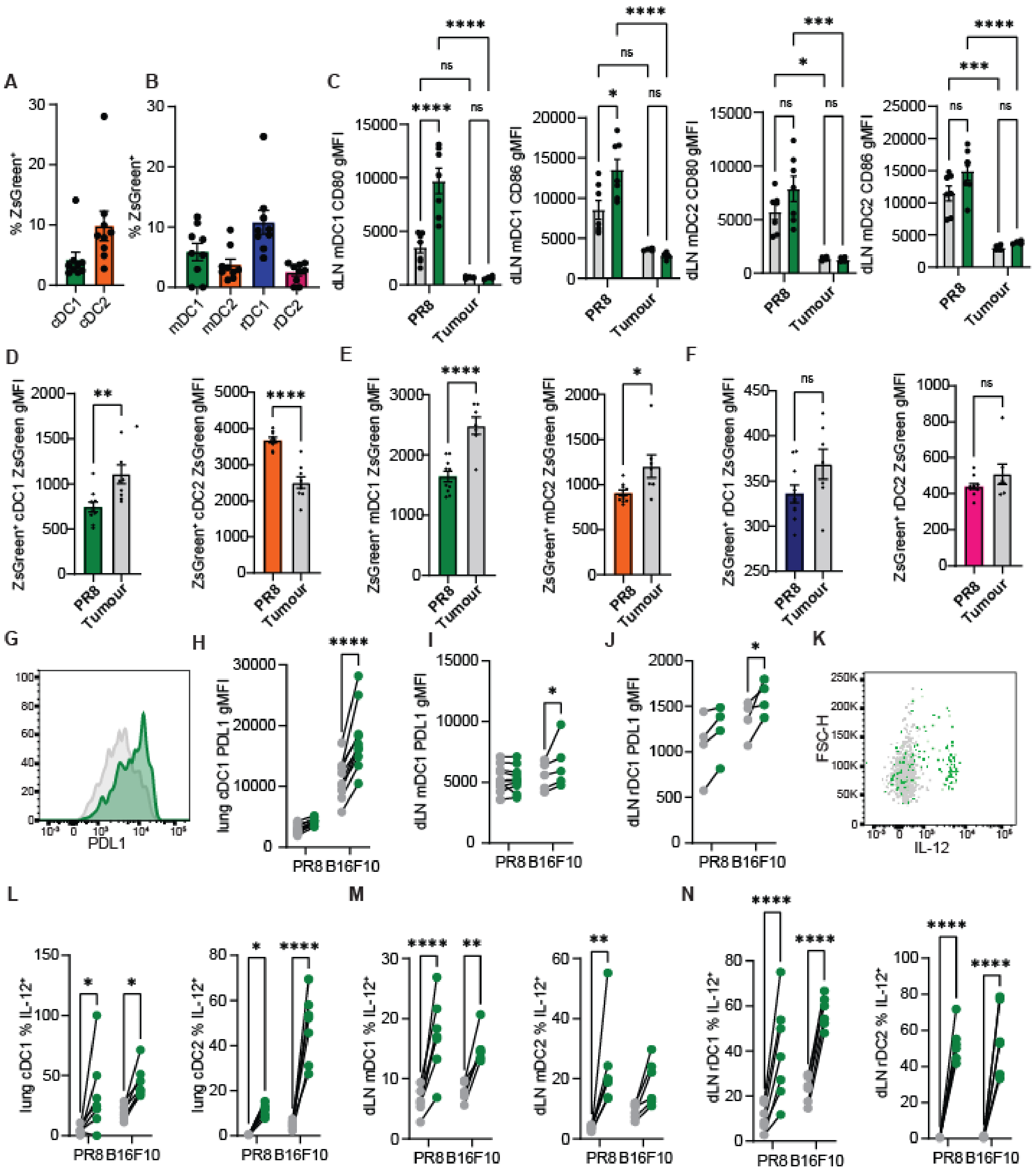

Fig. S7. Tumour antigen loaded rDC are differentially activated in BrightFlu and B16ZsGreen
Lungs and medLN were harvested from mice at d6 post infection with BrightFlu or at d14 post i.v. injection of B16ZsGreen. (A-B) ZsGreen loading was observed in lung (A) and LN (B) cDC subsets (n=9, 3 experiments) (C) CD80 and CD86 gMFI for mDC1 and mDC2 +/- ZsGreen was analysed by flow cytometry in the medLN (n=11, 3 experiments).(D-F) gMFI of ZsGreen in ZsGreen$^+$ cDC, was analysed by flow cytometry in the lung (D) and in the medLN in the mDC (E) and rDC (n=19, 3 experiments) (F).(G-J) gMFI of PD-L1 of ZsGreen$^+$ and ZsGreen$^-$ cDC, was analysed by flow cytometry, representative flow plot of ZsGreen$^+$ cells (green) and ZsGreen$^-$ cells (grey) (G). Quantification of gMFI in the lung (H) and in the medLN in the mDC (I) and rDC (n=20, 3 experiments) (J) (K-N) The percent of cDC1 +/- ZsGreen positive for IL-12 was analysed by flow cytometry. Representative flow plot for IL-12 stain in ZsGreen$^+$ (green) and ZsGreen$^-$ cDC (K). Quantification of IL-12 stain in the lung (L) and in the medLN in the mDC (M) and rDC

(N). Statistical differences were determined by Student's t-test. P values: ns $P>0.05$; *$P < 0.05$; **$P<0.01$; ***$P<0.001$ and ****$P < 0.0001$.

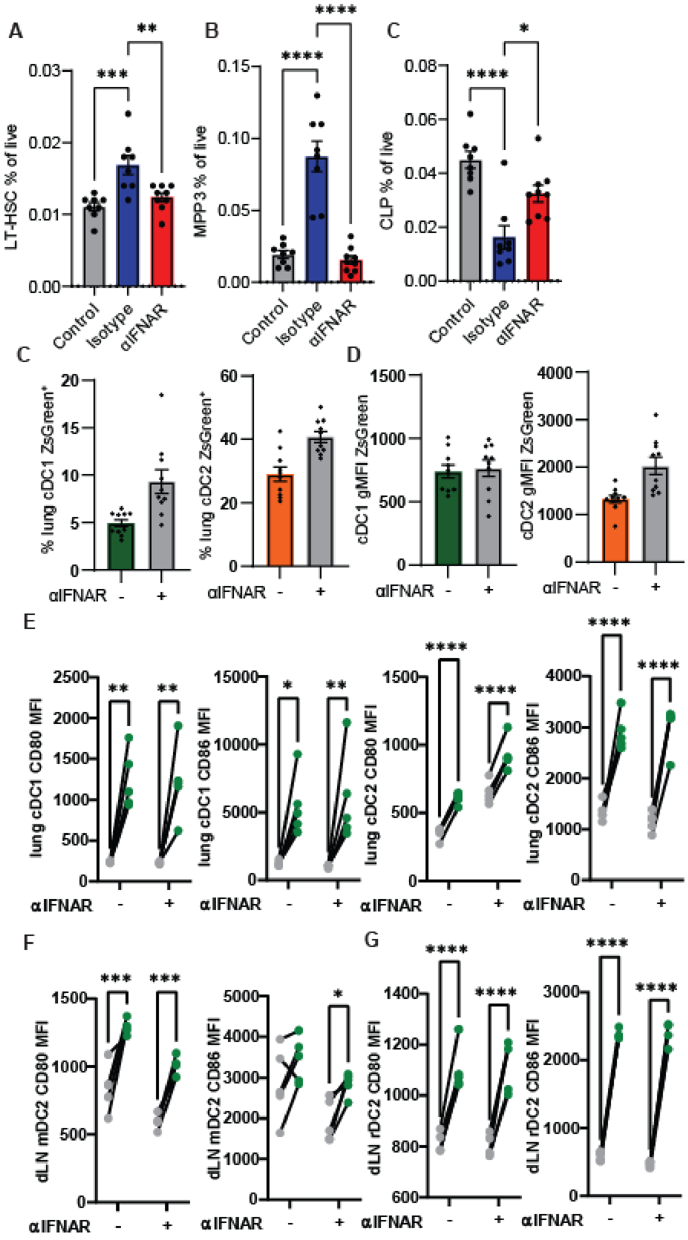

Fig. S8. Blockade of type I IFN signalling does not alter cDC activation in the lung or LN
(A-C) Bone marrow was harvested from mice at d6 post infection with BrightFlu with or without blocking antibody against the type I IFN receptor and the proportion of live cells made up of long-term haematopoietic stem cells (LT-HSC) (A), type 3 multipotent progenitors (MPP3) (B) and common lymphoid progenitors (CLP) (C) was assessed by flow cytometry (n=25, 3 experiments).(C-D) Lungs were harvested from mice at d6 post infection with BrightFlu in the presence of blocking antibody against type I IFN receptor or isotype control and the percentage of cDC subsets loaded with ZsGreen was analysed by flow cytometry (C) and the level of ZsGreen carried by loaded cDC was assessed by measuring gMFI of ZsGreen (n=20, 3 experiments)(D). (E-G) Lungs and medLN were harvested from mice at d6 post infection with BrightFlu in the presence of blocking antibody against type I IFN receptor or isotype control and the MFI of CD80 and CD86 was assessed by flow cytometry on antigen bearing and non-bearing cDC subsets in the lung (E) and in mDC2 (F) and rDC2 (G) within the medLN (n=10, 3 experiments). Statistical differences were

determined by one-way ANOVA with Tukey post-test. P values: *P < 0.05; **P<0.01; ***P<0.001 and ****P < 0.0001. Data are shown as mean ± SEM

Antibody	Fluorescence	Species	Clone	Catalogue number	Company
CD45	AF700	mouse	30-F11	103128	Biolegend
CD45.1	Percp5.5	mouse	A20	110728	Biolegend
CD45.2	BV421	mouse	104	109832	Biolegend
CD11B	BV605	mouse	M1/70	101257	Biolegend
CD11B	BV650	mouse	M1/70	101259	Biolegend
CD11B	BV421	mouse	M1/70	101236	Biolegend
CD11C	BV650	mouse	N418	117339	Biolegend
IL-12/IL-23	Percp5.5	mouse	C15.6	505211	Biolegend
LY6C	BV711	mouse	HK1.4	128037	Biolegend
LY6C	BV785	mouse	HK1.4	128041	Biolegend
LY6C	AF700	mouse	HK1.4	128023	Biolegend
LY6G	BV785	mouse	1A8	127645	Biolegend
Siglec-F	BV786	mouse	E50-2440	740956	BD
MHCII	AF700	mouse	M5/114.15.2	107622	Biolegend
MHCII	BV421	mouse	M5/114.15.2	107632	Biolegend
CD24	PECY7	mouse	M1/69	101822	Biolegend
CD103	APC	mouse	2E7	121414	Biolegend
CD103	Percp5.5	mouse	2E	121416	Biolegend
Epcam	BV650	mouse	G8.8	118241	Biolegend
CD31	APC	mouse	390	102410	Biolegend
B220	BV785	mouse	RA3-6B2	103224	Biolegend
CD90.2	BV785	mouse	30-H12	105331	Biolegend
CD3	BV785	mouse	145-2C11	100355	Biolegend
CD3	AF700	mouse	17A2	100216	Biolegend
CD4	AF647	mouse	RM4-5	100530	Biolegend
CD8	BV605	mouse	53-6.7	100743	Biolegend
CD80	PE	mouse	16-10A1	104708	Biolegend
CD86	BV510	mouse	GL-1	105040	Biolegend
CD40	Percp5.5	mouse	3/23	124623	Biolegend
CCR7	PE	mouse	4B12	120105	Biolegend
PDL1	BV711	mouse	10F.9G2	124319	Biolegend
Zombie	Nir™ Dye			77184	Biolegend

Table. S1. List of used antibodies
